# Unmasking herd protection by an oral cholera vaccine in a cluster-randomized trial

**DOI:** 10.1093/ije/dyz060

**Published:** 2019-04-09

**Authors:** Mohammad Ali, Firdausi Qadri, Deok Ryun Kim, Taufiqul Islam, Justin Im, Faisal Ahmmed, Yun Chon, Ashraful Islam Khan, Khalequ Zaman, Florian Marks, John D Clemens

**Affiliations:** 1 International Health, Johns Hopkins Bloomberg School of Public Health, Baltimore, MD, USA; 2 Infectious Diseases Division, International Centre for Diarrhoeal Disease Research, Bangladesh, Dhaka, Bangladesh; 3 Development & Delivery Unit, International Vaccine Institute, Seoul, Republic of Korea; 4 Department of Medicine, University of Cambridge, Cambridge, UK; 5 Department of Epidemiology, UCLA Fielding School of Public Health, Los Angeles, CA, USA

**Keywords:** Cholera, cluster-randomized trial, vaccine effectiveness, fried-egg design

## Abstract

**Background:**

Several studies have shown that inactivated, whole-cell oral cholera vaccines (OCVs) confer both direct protection on vaccinees and herd protection on populations. Because our earlier cluster-randomized effectiveness trial (CRT) in urban Bangladesh failed to detect OCV herd protection, we reanalysed the trial to assess whether herd effects were masked in our original analysis.

**Methods:**

A total of 267 270 persons were randomized to 90 approximately equal-sized clusters. In 60 clusters persons aged 1 year and older were eligible to receive OCV and in 30 clusters persons received no intervention and served as controls. We analysed OCV protection against severely dehydrating cholera for the entire clusters, as in our original analysis, and for subclusters consisting of residents of innermost households. We hypothesized that if OCV herd protection was attenuated by cholera transmission into the clusters from the outside in this densely populated setting, herd protection would be most evident in the innermost households.

**Results:**

During 2 years of follow-up of all residents of the clusters, total protection (protection of OCV recipients relative to control residents) was 58% [95% confidence interval (CI): 43%, 70%; *P*<0.0001], indirect protection (protection of non-OCV recipients in OCV clusters relative to control participants) was 16% (95% CI: –20%, 41%; *P*=0.35) and overall OCV protection (protection of all residents in the OCV clusters relative to control residents) was 46% (95% CI: 30%, 59%; *P*<0.0001). Analyses of the inner 75% and 50% households of the clusters showed similar findings. However, total protection was 75% (95% CI: 50%, 87%, *P*<0.0001), indirect protection 52% (95% CI: –9%, 79%; *P*=0.08) and overall protection 72% (95% CI: 49%, 84%; *P*<0.0001) for the innermost 25% households.

**Conclusion:**

Consistent with past studies, substantial OCV herd protective effects were identified, but were unmasked only by analysing innermost households of the clusters. Caution is needed in defining clusters for analysis of vaccine herd effects in CRTs of vaccines.

**Key Messages**

- In our earlier analysis of a cluster-randomized trial (CRT), we observed moderate total vaccine protection (protection of vaccinees owing both to direct vaccine protection and reduced transmission due to vaccination of the surrounding population) against severely dehydrating cholera.
- We wondered whether our conventional analysis of the CRT, in which participants in entire clusters were evaluated, had obscured actual oral cholera vaccine (OCV) herd protection due to transmission of cholera into clusters from the outside in this dense urban setting.
- We reanalysed the data using an approach called ‘fried-egg analysis’, in which only the innermost residents (‘yolks’) of the clusters were evaluated for total protection, indirect protection and overall protection by OCV against severely dehydrating cholera during 2 years of follow-up.
- Programmatically, the findings for the innermost clusters confirm the results of earlier studies indicating the OCVs confer herd protection, and extend these findings by showing that OCV can confer herd protection also in densely populated, poor urban settings with endemic cholera.
- Methodologically, the results caution that spuriously negative findings for vaccine herd protection can occur in CRTs of vaccines in which clusters are not insulated from transmission from the outside, and argue for care in the selection and analysis of clusters in these studies.

## Background

Population-level protection by vaccines, including not only direct protection of vaccinees but also vaccine herd protection of both vaccinees and non-vaccinees, is recognized to be critical to the public health impact and cost-effectiveness of the inactivated, whole-cell based oral cholera vaccines (OCVs).^1–5^ Two of these vaccines are now available in the global stockpile of oral cholera vaccines managed by the International Coordinating Group on Vaccine Provision (http://www.who.int/csr/disease/icg/qa/en/), consisting of members from the Red Cross, Médecins sans Frontières, the World Health Organization and UNICEF. In 2011 we initiated a large-scale, cluster-randomized effectiveness trial (CRT) of the OCV Shanchol^TM^ (Shanta Biotechnics-Sanofi, Hyderabad, India) in a poor urban slum population in Dhaka, Bangladesh, where cholera is hyperendemic. A CRT design was selected in order to evaluate the population-level, herd protective effects of this vaccine, since the CRT design offers a powerful approach to assessing these protective effects in an unbiased fashion.^5^^,^^6^ In contrast to a past CRT of the same OCV,^7^^,^^8^ however, our CRT revealed lower than expected levels of total vaccine protection (direct protection of vaccinees plus additional protection of vaccinees by vaccine herd effects), perhaps due to limited vaccine herd protection. Absent vaccine herd protection was later confirmed in additional unpublished analyses of the trial, a result that contrasted with several past studies of herd protection by OCVs.^1–3^

We wondered whether these surprising findings were real, perhaps related to the very high level of transmission of cholera in this densely populated, poor urban setting, or could possibly have been an artifact of the design of the study, especially the demarcation of clusters in dense urban slums in which considerable transmission of cholera to persons inside the clusters may have originated from the outside. Such transmission would be predicted to attenuate the level of measured vaccine herd protection.^9^^,^^10^ To address this possibility, we reanalysed OCV protection against the primary outcome of the trial, severely dehydrating cholera, using an analytic strategy that has been called the ‘fried-egg’ approach.^11^ With this approach, the analysis is confined to persons residing in the central ‘yolk’ of the cluster rather than the entire cluster, with the assumption that the persons residing in the central yolk are protected against cholera originating outside of the cluster, due to a buffering effect of persons residing in the outer ‘white’ of the cluster, who act as a barrier to such transmission. We hypothesized that as the ‘yolk’ of the cluster is progressively constricted to central populations residing further and further from the outer perimeter of the clusters, vaccine herd protective effects should become more apparent. Herein, we report the findings of this reanalysis.

## Methods

### The study area and population

We reanalysed the data of CRT of the effectiveness of the OCV that was conducted in an urban slum area of Dhaka, Bangladesh.^8^ This trial was designed to evaluate the population-level protective effects of this vaccine when administered under programmatic conditions and to assess whether a behavioural intervention to improve drinking-water quality and handwashing (WASH) practices added to the protection conferred by the OCV. Before vaccination, a census was carried out to register the study area population. Information was collected using personal digital assistants (PDAs) from households after verbal consent of the respondents. A unique identification number was given to each registered individual and relevant socio-economic, demographic and healthcare information was collected. In this CRT residents of 90 geographic clusters in urban Dhaka were randomized to one of three arms: OCV alone, OCV combined with the WASH behavioural change intervention, or no intervention. There was a buffer zone of at least 30 meters between clusters to minimize the spillover of the effect of behavioural intervention. The 90 clusters, with a total population of 267 270, were randomly assigned to the different arms of the study in blocks of three, after stratifying the clusters by distance of the cluster centroid to the nearest study hospital (lower than the median distance vs the median distance or higher), yielding 30 clusters in each arm with similar numbers of residents in each of the three trial arms. The average cluster population size was 2988 (range 2288–4299). For the purpose of this analysis, we combined the two intervention arms into one arm of 60 clusters, as the WASH intervention was found to add little protection against cholera to the OCV and we wished to increase the statistical power of our analysis.^8^

### Eligibility and interventions

Zero time was defined as the date of the first dose for vaccinees; median date of the first dose in the cluster for non-vaccinees in the intervention clusters, and for residents of the non-intervention clusters, it was the median date of the first dose among residents of the nearest intervention cluster. Consenting, non-pregnant residents of clusters of the two intervention arms were eligible to receive the OCV if they were 1 year of age or older at zero time. The behavioural change WASH intervention, which is described in detail elsewhere, was targeted to all households in the clusters of the arm receiving both OCV and WASH.^8^ OCV was given as a two dose regimen delivered at fixed vaccination sites, with an interdose interval of at least 14 days. Vaccination was conducted between 17 February and 16 April, 2011.

### Diarrhoea surveillance and definitions

Surveillance for diarrhoea was conducted at all 12 hospitals known to provide care for diarrhoea to the study population. Patients from the study area were identified by use of household identification cards and/or an on-site computerized database. Clinical data were recorded onto structured forms after obtaining written informed consent from the patient or the guardian of the patient in case of a minor. A fecal specimen was taken from every diarrhoea patient and transported to the icddr,b (International Centre For Diarrhoeal Disease Research, Bangadesh) laboratory for carrying out microbiological tests for *Vibrio cholerae* O1 and O139, and enterotoxigenic *Escherichia coli*, as described elsewhere.^12–15^ A diarrhoeal visit was defined as having 3 or more loose motions in the 24 hours before presentation or 1–2 or an indeterminate number of loose stools with evidence of dehydration according to WHO criteria.^16^ Diarrhoeal visits for which the date of onset was ≤7 days from the date of discharge for the previous visit were grouped into the same diarrhoeal episode. The onset of a diarrhoeal episode was the day of onset of symptoms reported in the first diarrhoeal visit of the episode. A severely dehydrating cholera case was defined as a diarrhoeal episode in which severe dehydration by WHO criteria was noted in at least one constituent visit, there was no passage of bloody stools in any constituent visit, a fecal specimen yielded *V. cholerae* O1 or O139 in at least one constituent visit, and a domiciliary check confirmed that the patient had indeed visited the treatment centre for care of diarrhoea on the recorded dates of visits for the episode. An Enterotoxigenic Escherichia coli (ETEC) diarrhoeal episode was defined as a non-bloody diarrhoeal episode in which a faecal specimen yielded ETEC, but specimens in all component visits did not yield *V. cholerae* O1 or O139.

### Defining the yolk for the fried-egg analytic approach

**Figure 1.**
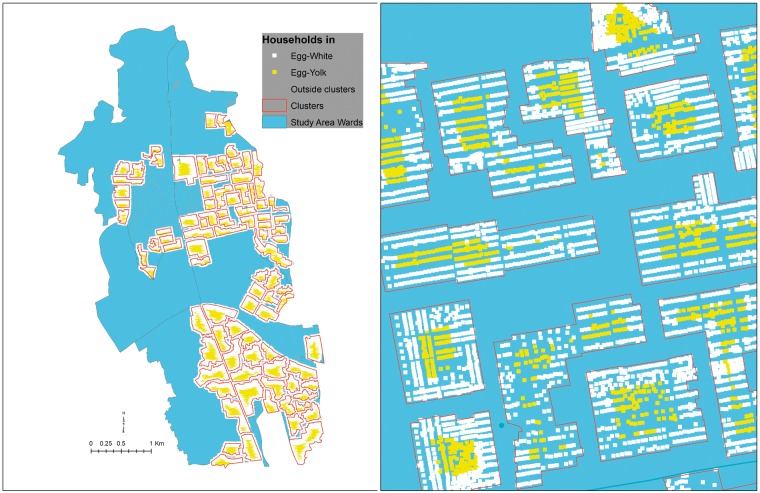
Distribution of study area households (entire study area in the left panel, close-up view of some of the clusters in the right panel) for analyses of the P25 clusters.

We used the ‘fried-egg’ approach to reanalyse the data for this trial.^11^ We analysed OCV protection for the entire clusters, as well as for residents of the innermost 75%, innermost 50% or innermost 25% households (‘yolks’) of the clusters. We hypothesized that if herd protection was attenuated by transmission of cholera into the clusters from the outside, this protection would be most evident in the innermost households. To demarcate these different sized ‘yolks’, we calculated the linear distance to the nearest cluster perimeter for every household and sorted the households in each cluster in ascending (furthest to closest to the nearest perimeter) order by distance. We then assembled successive proportions of households, beginning with the household furthest from the perimeter and proceeding to include households progressively closer to the nearest perimeter, until the desired fraction of households was reached. Before undertaking the analysis, we specified four fractions of households for analysis: 25% (innermost yolk), 50%, 75% and 100% (outermost yolk including the entire cluster), referred to, respectively as P25, P50, P75 and P100. Figure 1 shows the selected households for the P25 group.

## Analysis

As in our original analysis of this trial, severely dehydrating cholera was the primary outcome of interest.^8^ We analysed all measures of OCV protection against severely dehydrating cholera as the proportionate reduction of disease incidence [(1-hazard ratio of severely dehydrating cholera in the intervention clusters vs the control clusters) x 100%]. For assessment of total OCV protection we compared vaccinees in the intervention clusters vs residents aged 1 year and older at zero time of the control clusters; for indirect OCV protection we compared all non-vaccinees in the intervention clusters vs all residents of the control clusters; and for overall OCV protection we compared all residents of the intervention clusters vs all residents of the control clusters.^6^ For overall and indirect protection, we analysed all age groups, including those too young to have been vaccinated. For total protection, we analysed only persons who would have been age-eligible for vaccination. We hypothesized that population-level OCV protection should become more pronounced the longer the distance of the household from the nearest perimeter. As in our earlier analysis,^8^ we considered all persons present at the time of the second dose, defined as the date of the second dose for vaccinees; the median date of the second dose for one-dose or non-vaccinees in the intervention clusters; and for the non-intervention clusters, the median date of the second dose among residents of the nearest intervention cluster. We analysed initial severely dehydrating cholera episodes occurring from 14 days to 2 years after the second dose. Similarly, we analysed initial ETEC episodes occurring from 14 days to 2 years after the second dose in a bias indicator analysis. This bias indicator analysis was undertaken because our strategy of analysing all controls, but only vaccinees and non-vaccinees in the intervention clusters for measurement of total and indirect protection, respectively, entailed non-randomized comparisons. We measured overall, total and indirect protection by the OCV against severely dehydrating cholera, redefining the clusters according to the P25, P50, P75 and P100 populations created with the fried-egg design.

We conducted survival analyses, censoring individuals who died or migrated out before the end of the follow-up period. In-migrants and infants born after zero time were not included in the analysis. Because there was little movement between the clusters, there was very little contamination of the control clusters with migrating vaccinees**.** We fitted Cox proportional hazards regression models after adjusting for age at zero time, sex, and the stratification variable (distance to the hospital) used for cluster randomization, as well as variables found to be unequally distributed at *P* < 0 .05 in bivariate, baseline comparisons of the arms of the study for each concentric zone and each type of population-level OCV protection. We ascertained that the proportional hazards assumption was fulfilled for each fitted independent variable in the models. We used robust sandwich variance estimates to account for the design effect of cluster randomization, allowing inferences about vaccine protection at the individual level, and we estimated the hazard ratios by exponentiation of the coefficient for the treatment arm variable in these models.^17^ The 95% confidence intervals (95% CIs) and *P*-values were calculated two-tailed, considering *P* < 0.05 as the threshold of statistical significance. All statistical analyses were performed using SAS version 9.4.

### Ethical considerations

The study protocol was approved by the Research Review Committee and the Ethical Review Committee of the icddr, b, Dhaka, Bangladesh and the Institutional Review Board of the International Vaccine Institute. Written informed consent was obtained from residents 18 years and older and from the parents or guardians of residents aged 1–17 years of age during vaccination. Additional assent was obtained from residents aged 11–17 years. The study protocol was registered at ClinicalTrials.gov number, NCT01339845.

### Role of the funding source

The study was funded by the Bill and Melinda Gates Foundation (OPP1171432). The funder had no role in data analysis, data interpretation or writing of this manuscript. The corresponding author had full access to all the data in the study and had final responsibility for the decision to submit the manuscript for publication.

## Results

The assembly of subjects and outcomes for analysis has been published previously.^8^ The intervention and non-intervention groups under analysis for the analysis of overall, total and indirect OCV protection were comparable with respect to distributions of sociodemographic characteristics at zero time except for a few variables (Tables 1–4 and Supplementary Tables S1–S8, available as Supplementary data at *IJE* online). For analysis of overall OCV protection against severely dehydrating cholera, there were 267 270 individuals and 226 cholera episodes in the P100 group (Table 5); 205 313 persons and 175 cholera episodes in the P75 group; 136 068 persons and 117 episodes in the P50 group, and 66 473 persons and 49 cholera episodes in the P25 group. Correspondingly, overall protection was 46% (95% CI: 30–59%; *P*-value <0.0001) for the P100 group, with similar values for the P75 and P50 groups, but 72% (95% CI: 49–84%; *P*-value <0.0001) for the P25 group (Table 5).

**Table 1.** Sociodemographic characteristics of the individuals in the vaccine and non-vaccine arms of the study in analysis of the P100 clusters for total vaccine protection

Variables	Intervention arm (*n* = 123, 659)	Non-intervention arm (*n* = 78, 518)	*P* value^a^
Age at zero time – years^b^	23.22±15.75	24.58±15.77	<.0001
Male sex - no. (%)	56 196 (45.44)	38 485 (49.01)	<.0001
Living in own house - no. (%)	28 011 (22.65)	19 714 (25.11)	0.72
Living in the study area less than 1 year - no. (%)	51 903 (41.97)	31 725 (40.40)	0.67
Living in household^f^ sharing kitchen with others - no. (%)	108 766 (87.96)	65 258 (83.11)	0.32
Living in household with improved water source - no. (%)^c^	6 807 (5.50)	4 127 (5.26)	0.73
Living in household using treated water for drinking - no. (%)^d^	68 138 (55.10)	41 457 (52.80)	0.68
Living in household using fixed place for waste disposal- no. (%)	101 172 (81.82)	60 744 (77.36)	0.36
Living in household having concrete roof - no. (%)	16 194 (13.10)	12 269 (15.63)	0.36
Living in house with sanitary toilet - no. (%)	723 (0.58)	343 (0.44)	0.56
Monthly expenditure of household - Bangladesh taka^e^	10 027±4637	9790±4562	0.31

a The *P* values were derived by comparing the differences between the two groups adjusted for cluster effects using generalized estimating equations with the logit link function for dichotomous variables and the identity link function for dimensional variables.

b Zero time was defined as the date of dose 1 for vaccinees, and at the median date of dose 1 of the cycle of vaccination in the clusters for non-vaccinees.

c An improved water source was defined as own tap.

d Water that was boiled, filtered, or chlorinated was considered to have been treated.

e One US dollar equals approximately 80 Bangladeshi taka.

f A household was defined as residents living in a compound and sharing the same cooking pot.

Note: ± values are means±SD.

**Table 2.** Sociodemographic characteristics of the individuals in the vaccine and non-vaccine arms of the study in analysis of the P25 clusters for total vaccine protection

Variables	Intervention arm (*n* = 30 342)	Non-intervention arm (*n* = 20 343)	*P* value^a^
Age at zero time - years^b^	23±15.81	25±15.77	<.0001
Male sex - no. (%)	13 789 (45.45)	9989 (49.10)	<.0001
Living in own house - no. (%)	7356 (24.24)	4885 (24.01)	0.98
Living in the study area less than 1 year - no. (%)	12 254 (40.39)	8648 (42.51)	0.74
Living in household^f^ sharing kitchen with others - no. (%)	26 595 (87.65)	17 256 (84.83)	0.49
Living in household with improved water source - no. (%)^c^	1511 (4.98)	944 (4.64)	0.76
Living in household using treated water for drinking - no. (%)^d^	16 963 (55.91)	10 333 (50.79)	0.41
Living in household using fixed place for waste disposal - no. (%)	25 226 (83.14)	15 563 (76.50)	0.34
Living in household having concrete roof - no. (%)	4283 (14.12)	2929 (14.40)	0.74
Living in house with sanitary toilet - no. (%)	180 (0.59)	68 (0.33)	0.33
Monthly expenditure of household - Bangladesh taka^e^	10 109±4709	9680±4048	0.11

a The *P* values were derived by comparing the differences between the two groups adjusted for cluster effects using generalized estimating equation with the logit link function for dichotomous variables and the identity link function for dimensional variables.

b Zero time was defined as the date of dose 1 for vaccinees, and at the median date of dose 1 of the cycle of vaccination in the clusters for non-vaccinees.

c An improved water source was defined as own tap.

d Water that was boiled, filtered, or chlorinated was considered to have been treated.

e One US dollar equals approximately 80 Bangladeshi taka.

f A household was defined as residents living in a compound and sharing the same cooking pot.

Note: ± values are means±SD.

**Table 3.** Sociodemographic characteristics of the individuals in the vaccine and non-vaccine arms of the study in analysis of the P100 clusters for indirect vaccine protection

Variables	Intervention arm (*n* = 45 784)	Non-intervention arm (*n* = 80 056)	*P* value^a^
Age at zero time - years^b^	25.11 + 15.83	24.10 + 15.98	<.0001
Male sex - no. (%)	25 182 (55.00)	39 264 (49.05)	<.0001
Living in own house - no. (%)	8222 (17.96)	20 075 (25.08)	0.26
Living in the study area less than 1 year - no. (%)	23 776 (51.93)	32 424 (40.50)	0.0003
Living in household^f^ sharing kitchen with others - no. (%)	40 813 (89.14)	66 536 (83.11)	0.27
Living in household with improved water source - no. (%)^c^	2105 (4.60)	4197 (5.24)	0.78
Living in household using treated water for drinking - no. (%)^d^	25 012 (54.63)	42 276 (52.81)	0.85
Living in household using fixed place for waste disposal- no. (%)	38 141 (83.31)	61 943 (77.37)	0.26
Living in household having concrete roof - no. (%)	7390 (16.14)	12 524 (15.64)	0.98
Living in house with sanitary toilet - no. (%)	286 (0.62)	352 (0.44)	0.53
Monthly expenditure of household - Bangladesh taka^e^	9 923 + 4, 962	9 773 + 4, 548	0.73

a The *P* values were derived by comparing the differences between the two groups adjusted for cluster effects using generalized estimating equation with the logit link function for dichotomous variables and the identity link function for dimensional variables.

b Zero time was defined as the date of dose 1 for vaccinees, and at the median date of dose 1 of the cycle of vaccination in the clusters for non-vaccinees.

c An improved water source was defined as own tap.

d Water that was boiled, filtered, or chlorinated was considered to have been treated.

e One US dollar equals approximately 80 Bangladeshi taka.

f A household was defined as residents living in a compound and sharing the same cooking pot.

Note: ± values are means±SD.

**Table 4.** Sociodemographic characteristics of the individuals in the vaccine and non-vaccine arms of the study in analysis of the P25 clusters for indirect vaccine protection

Variables	Intervention arm (*n* = 11 192)	Non-intervention arm (*n* = 20 725)	*P* value^a^
Age at zero time – years^b^	25.07±15.70	24.15±15.97	<.0001
Male sex - no. (%)	6102 (54.52)	10 176 (49.10)	<.0001
Living in own house - no. (%)	2012 (17.98)	4962 (23.94)	0.40
Living in the study area less than 1 year - no. (%)	5708 (51.00)	8843 (42.67)	0.02
Living in household^f^ sharing kitchen with others - no. (%)	9990 (89.26)	17 588 (84.86)	0.41
Living in household with improved water source - no. (%)^c^	471 (4.21)	960 (4.63)	0.74
Living in household using treated water for drinking - no. (%)^d^	6291 (56.21)	10 537 (50.84)	0.59
Living in household using fixed place for waste disposal- no. (%)	9523 (85.09)	15 852 (76.49)	0.25
Living in household having concrete roof - no. (%)	1973 (17.63)	2994 (14.45)	0.72
Living in house with sanitary toilet - no. (%)	70 (0.63)	70 (0.34)	0.20
Monthly expenditure of household – Bangladesh taka^e^	10 053±5054	9660±4034	0.37

a The *P* values were derived by comparing the differences between the two groups adjusted for cluster effects using generalized estimating equation with the logit link function for dichotomous variables and the identity link function for dimensional variables.

b Zero time was defined as the date of dose 1 for vaccinees, and at the median date of dose 1 of the cycle of vaccination in the clusters for non-vaccinees.

c An improved water source was defined as own tap.

d Water that was boiled, filtered, or chlorinated was considered to have been treated.

e One US dollar equals approximately 80 Bangladeshi taka.

f A household was defined as residents living in a compound and sharing the same cooking pot.

Note: ± values are means±SD.

**Table 5.** Overall, total and indirect OCV protection against severely dehydrating cholera in the differently defined clusters

Measures of protection	Intervention arm (no. of clusters = 60)			Non-intervention arm (no. of clusters = 30)			Protective effectiveness (PE)^a^		
	No. of persons	No. of cases	Rate/1000 person-years	No. of persons	No. of cases	Rate/1000 person-years	PE (%)	95% CI	*P* value
P100 clusters									
Overall	187 214	120	0.53	80 056	106	0.98	46	30 to 59	<0.0001
Total	123 659	64	0.41	78 518	105	0.99	58	43 to 70	<0.0001
Indirect	45 784	43	0.83	80 056	106	0.98	16	−20 to 41	0.3502
P75 clusters									
Overall	143 915	97	0.56	61 398	78	0.95	41	21 to 56	0.0005
Total	95 254	56	0.46	60 196	77	0.95	51	30 to 65	<0.0001
Indirect^b^	35 039	32	0.81	61 398	78	0.95	12	−31 to 43	0.5000
P50 clusters									
Overall	95 310	62	0.54	40 758	55	1.00	47	23 to 63	0.0007
Total	63 185	31	0.38	39 960	55	1.02	62	40 to 75	<0.0001
Indirect^b^	23 156	25	0.95	40 758	55	1.00	1	−60 to 39	0.9557
P25 clusters									
Overall	45 748	18	0.32	20 725	31	1.13	72	49 to 84	<0.0001
Total	30 342	11	0.28	20 343	31	1.16	75	50 to 87	<0.0001
Indirect	11 192	7	0.56	20 725	31	1.13	52	−9 to 79	0.0783

Note: for overall and indirect protection, we analysed all age groups, including those too young to have been vaccinated. For total protection, we analysed only persons who would have been age-eligible for vaccination.

a Adjusted for age at zero time (described in the text), sex, stratification variable (distance to the hospital) for cluster randomization, and the variables found to be significantly different (*P* < 0.05) between the arms at baseline.

b In addition to age, sex and distance to the hospital, the variable ‘living in the study area <1 year’ was adjusted for in analyses of indirect vaccine protection of the P75 clusters. Because of the small number of outcome events for the P25 group, this variable was not adjusted for in analysis of indirect vaccine protection in this group.

Similarly, analysis of total OCV protection in the P100 group yielded an estimate of 58% (95% CI: 43–70%; *P*-value <0.0001) (Table 5), with similar estimates for the P75 and P50 groups, but rose to 75% (95% CI: 50–87%; *P*-value <0.0001) when the analysed clusters were restricted to the P25 group. Likewise, indirect OCV protection was estimated at 16% (95% CI: –20–41%; *P*-value =0.35) in the P100 group, but rose to 52% (95% CI: –9–79%; *P*-value =0.08) in the P25 group (Table 5).

Finally, we reasoned that the results of our analyses would be strengthened if we failed to find a similar pattern of OCV protection for a clinically similar outcome that OCV was not anticipated to prevent, ETEC diarrhoea. The analyses showed no overall, total or indirect OCV protection against ETEC diarrhoea in the P100 group as well as in the P75, P50 and P25 groups (Table 6).

**Table 6.** Overall, total and indirect protection against ETEC diarrhoea in the differently defined clusters

Measures of protection	Intervention arm (no. of clusters = 60)			Non-intervention arm (no. of clusters = 30)			Protective effectiveness (PE)^a^		
	No. of persons	No. of cases	Rate/1000 person-years	No. of persons	No. of cases	Rate/1000 person-years	PE (%)	95% CI	*P* value
P100 clusters									
Overall	187 214	277	1.23	80 056	140	1.30	6	–16 to 23	0.5819
Total	123 659	153	0.98	78 518	110	1.04	5	–21 to 26	0.6738
Indirect	45 784	106	2.05	80 056	140	1.30	–65	–113 to –28	<0.0001
P75 clusters									
Overall	143 915	213	1.22	61 398	103	1.25	2	–24 to 23	0.8532
Total	95 254	120	0.99	60 196	81	1.00	1	–33 to 24	0.9690
Indirect^b^	35 039	81	2.05	61 398	103	1.25	–70	–127 to –26	0.0004
P50 clusters									
Overall	95 310	153	1.32	40 758	69	1.26	–5	–40 to 21	0.7309
Total	63 185	82	1.02	39 960	55	1.02	0	–42 to 28	0.9574
Indirect^b^	23 156	61	2.33	40 758	69	1.26	–89	–168 to –34	0.0003
P25 clusters									
Overall	45 748	69	1.24	20 725	36	1.32	6	–41 to 37	0.7651
Total	30 342	38	0.98	20 343	32	1.19	13	–33 to 48	0.4320
Indirect^b^	11 192	25	1.99	20 725	36	1.32	–55	–158 to 7	0.0953

Note: for overall and indirect protection, we analysed all age groups, including those too young to have been vaccinated. For total protection, we analysed only persons who would have been age-eligible for vaccination.

a Adjusted for age at zero time (described in the text), sex, stratification variable (distance to the hospital) for cluster randomization, and the variables found to be significantly different (*P* < 0.05) between the arms at baseline.

b In addition to age, sex, and distance to the hospital, the variable ‘living in the study area less than <1 year’ was adjusted for in analyses of indirect vaccine protection.

## Discussion

Our reanalysis of this CRT suggests that the analysis of entire clusters for the trial, as we previously reported,^8^ gave a false impression that population-level vaccine herd effects were insignificant when the OCV was given in this dense urban setting. Indeed, restriction of the analyses to the central 25% of the clusters revealed substantial levels of overall, total and indirect vaccine protection against severely dehydrating cholera, although the estimate of indirect protection was unstable, reflected by a wide sample CI. To our knowledge, this is the first study to demonstrate the impact of using the fried-egg analytic approach on levels of population-level vaccine protection against an infectious disease studied in a CRT.

Before discussing the implications of this analysis, it is important to address several potential limitations. Because of the design of the fried-egg analysis, our results refer to OCV herd protection of a population that is also surrounded by a vaccinated population. In this situation, herd protection could have been conferred not only by vaccination of the central ‘yolk’ under analysis, but also by the surrounding vaccinated population. However, we do not consider this a significant limitation of our analysis, since this observation does not detract from the validity of the conclusion that OCV herd protection was observed in this densely populated, urban setting. Moreover, since OCV is usually delivered in mass immunization programmes covering a wide population, a significant fraction of targeted populations is typically surrounded by a rim of vaccinated population in such settings. It might also be argued that because the control clusters in the trial did not receive a comparator agent, the estimates of total and indirect OCV protection might have been biased, since the risk of cholera might differ between participants and non-participants. Arguing against this possibility, however, is the observation that our analysis of a bias indicator condition, ETEC diarrhoea, against which OCV should have had no protective effect, gave no evidence of either of these types of herd protection. Indeed, our analyses found that the rate of ETEC diarrhoea was higher in the unvaccinated subgroup in the intervention clusters than that in all residents in the control clusters, suggesting a possible selection bias that would make our findings for OCV indirect protection conservative.

From a public health perspective, our results are consistent with other analyses of vaccine herd protection of OCVs, both in rural^2^ and urban–periurban^3^ cholera-endemic settings, which demonstrated substantial vaccine herd protection. Such herd protection greatly enhances the impact and cost-effectiveness of these vaccines.^4^ As well, our results suggest that if small clusters of a population are vaccinated with OCV in densely populated, urban, cholera-affected settings, a large proportion of members of the clusters may fail to be benefitted by herd effects, a problem that may be addressed by vaccinating large contiguous populations.

Our analyses also have implications for the design and analysis of future CRTs of vaccines intended to evaluate population-level vaccine herd protection. CRTs are acknowledged to be the best design for arriving at unbiased estimates of vaccine herd protective effects.^6^ However, a caveat of this assertion is that clusters are selected and defined in such a fashion that they are true epidemiological units of person-to-person transmission, meaning that no such transmission of the target pathogen occurs into the clusters from the surrounding populations. In practice, it will be uncommon that selected clusters will meet this idealized condition, and measured vaccine herd protective effects may be diluted to the extent to which the condition is violated.^9^ One approach that is commonly used in CRTs is to create buffer zones between clusters, as was done in this trial. However, as illustrated by our analysis, buffer zones, while potentially useful for minimizing diffusion of interventions between clusters, are not necessarily effective in preventing transmission of pathogens into clusters from the outside. Because it may be difficult to predict in advance whether such transmission will occur in a CRT of vaccines, some variant of the fried-egg design may be considered to determine inner cluster sizes for primary analyses of vaccine protection, or at least explored in secondary sensitivity analyses.

In conclusion, our reanalysis of this trial revealed high levels of OCV herd protection in innermost populations of clusters, which had been obscured in analyses of entire clusters. These data should provide reassurance to policymakers regarding the use of OCVs in densely populated, poor urban settings, where the force of cholera infection may be high. Our findings also sound a note of caution that CRTs of vaccines against infections transmitted from person to person in which clusters fail to fulfill epidemiological assumptions about transmission of the target pathogen may underestimate vaccine herd effects.

## Funding

The work was supported by the Bill & Melinda Gates Foundation (OPP1171432). The study was also supported by core grants to the icddr, b from the Governments of Bangladesh, Canada, Sweden, and the UK. The funders had no role in study design, data collection and analysis, decision to publish or preparation of the manuscript.

## Supplementary Material

dyz060_Supplementary_Data
