# Supplementary material for: Unmasking herd protection by an oral cholera vaccine in a cluster-randomized trial
Source: Int J Epidemiol. 2019 Apr 9;48(4):1252–61. doi: 10.1093/ije/dyz060 (PMC6693801; doi:10.1093/ije/dyz060)
Supplement: dyz060_Supplementary_Data [file dyz060_supplementary_data.docx]

Table S1. Sociodemographic characteristics of the individuals in the vaccine and non-vaccine arms of the study in analysis of the P100 clusters for overall vaccine protection

| Variables | Intervention Arm  (n=187,214) | Non-intervention arm  (n=80,056) | P value* |
| --- | --- | --- | --- |
| Age at zero time – years† | 24±15.71 | 24±15.98 | 0.15 |
| Male sex - no. (%) | 90,841 (48.52) | 39,264 (49.05) | 0.02 |
| Living in own house - no. (%) | 38,837 (20.74) | 20,075 (25.08) | 0.52 |
| Living in the study area less than one year - no.(%) | 85,278 (45.55) | 32,424 (40.50) | 0.13 |
| Living in household** sharing kitchen with others - no. (%) | 165,693 (88.50) | 66,536 (83.11) | 0.29 |
| Living in household with improved water source - no. (%)‡ | 9,543 (5.10) | 4197 (5.24) | 0.95 |
| Living in household using treated water for drinking - no. (%)§ | 102,037 (54.50) | 42,276 (52.81) | 0.76 |
| Living in household using fixed place for waste disposal- no. (%) | 153,780 (82.14) | 61,943 (77.37) | 0.33 |
| Living in household having concrete roof - no. (%) | 25,712 (13.73) | 12,524 (15.64) | 0.49 |
| Living in house with sanitary toilet - no. (%) | 1145 (0.61) | 352 (0.44) | 0.88 |
| Monthly expenditure of household – Bangladesh taka¶ | 9940 ± (4684) | 9773± (4548) | 0.49 |

* The P values were derived by comparing the differences between the two groups adjusted for cluster effects using generalized estimating equations with the logit link function for dichotomous variables and the identity link function for dimensional variables

†Zero time was defined as the date of dose 1 for vaccinees, and at the median date of dose 1 of the cycle of vaccination in the clusters for non-vaccinees

‡ An improved water source was defined as own tap

§ Water that was boiled, filtered, or chlorinated was considered to have been treated

¶One U.S. dollar equals approximately 80 Bangladeshi taka

** A household was defined as residents living in a compound and sharing the same cooking pot.

Note: Plus–minus values are means±SD

Table S2. Sociodemographic characteristics of the individuals in the vaccine and non-vaccine arms of the study in analysis of the P75 clusters for overall vaccine protection

| Variables | Intervention Arm  (n=143,915) | Non-intervention arm (n=61,398) | P value* |
| --- | --- | --- | --- |
| Age at zero time – years† | 24±15.70 | 24±15.98 | 0.17 |
| Male sex - no. (%) | 69,746 (48.46) | 30,103 (49.03) | 0.02 |
| Living in own house - no. (%) | 29,983 (20.83) | 15,813 (25.75) | 0.46 |
| Living in the study area less than one year - no.(%) | 64,972 (45.15) | 24,866 (40.50) | 0.19 |
| Living in household** sharing kitchen with others - no. (%) | 127,523 (88.61) | 50,900 (82.90) | 0.28 |
| Living in household with improved water source - no. (%)‡ | 7,048 (4.90) | 31,86 (5.19) | 0.91 |
| Living in household using treated water for drinking - no. (%)§ | 78,116 (54.28) | 31,657 (51.56) | 0.68 |
| Living in household using fixed place for waste disposal- no. (%) | 118,408 (82.28) | 46985 (76.53) | 0.30 |
| Living in household having concrete roof - no. (%) | 20,270 (14.08) | 9,213 (15.01) | 0.70 |
| Living in house with sanitary toilet - no. (%) | 855 (0.59) | 255 (0.42) | 0.44 |
| Monthly expenditure of household – Bangladesh taka¶ | 9,927±3,998 | 9,657±3,998 | 0.29 |

* The p-values were derived by comparing the differences between the two groups adjusted for cluster effects using generalized estimating equations with the logit link function for dichotomous variables and the identity link function for dimensional variables

†Zero time was defined as the date of dose 1 for vaccinees, and at the median date of dose 1 of the cycle of vaccination in the clusters for non-vaccinees

‡ An improved water source was defined as own tap

§ Water that was boiled, filtered, or chlorinated was considered to have been treated

¶One U.S. dollar equals approximately 80 Bangladeshi taka

** A household was defined as residents living in a compound and sharing the same cooking pot.

Note: Plus–minus values are means±SD

Table S3. Sociodemographic characteristics of the individuals in the vaccine and non-vaccine arms of the study in analysis of the P50 clusters for overall vaccine protection

| Variables | Intervention arm (n=95,310) | Non-intervention arm (n=40,758) | P value* |
| --- | --- | --- | --- |
| Age at zero time – years† | 24±15.71 | 24±16.03 | 0.28 |
| Male sex - no. (%) | 46,234(48.51) | 20,000 (49.07) | 0.08 |
| Living in own house - no. (%) | 20,540(21.55) | 10,497 (25.75) | 0.57 |
| Living in the study area less than one year - no.(%) | 42,493(48.51) | 16,632 (40.81) | 0.30 |
| Living in household** sharing kitchen with others - no. (%) | 84,233 (88.38) | 34,031 (83.50) | 0.36 |
| Living in household with improved water source - no. (%)‡ | 4,591 (4.82) | 2,051 (5.03) | 0.96 |
| Living in household using treated water for drinking - no. (%)§ | 51,995(54.54) | 20,904 (51.29) | 0.63 |
| Living in household using fixed place for waste disposal- no. (%) | 79,070 (82.96) | 31,410 (77.06) | 0.30 |
| Living in household having concrete roof - no. (%) | 13,846 (14.53) | 5,946 (14.59) | 0.91 |
| Living in house with sanitary toilet - no. (%) | 639 (0.67) | 166 (0.41) | 0.39 |
| Monthly expenditure of household – Bangladesh taka¶ | 9,926±4,826 | 9,630±4,064 | 0.27 |

* The p-values were derived by comparing the differences between the two groups adjusted for cluster effects using generalized estimating equation with the logit link function for dichotomous variables and the identity link function for dimensional variables

†Zero time was defined as the date of dose 1 for vaccinees, and at the median date of dose 1 of the cycle of vaccination in the clusters for non-vaccinees

‡ An improved water source was defined as own tap

§ Water that was boiled, filtered, or chlorinated was considered to have been treated

¶One U.S. dollar equals approximately 80 Bangladeshi taka

** A household was defined as residents living in a compound and sharing the same cooking pot.

Note: Plus–minus values are means±SD

Table S4. Sociodemographic characteristics of the individuals in the vaccine and non-vaccine arms of the study in analysis of the P25 clusters for overall vaccine protection

| Variables | Intervention arm (n=45,748) | Intervention arm (n=20,725) | P value* |
| --- | --- | --- | --- |
| Age at zero time – years† | 24±15.70 | 24±15.97 | 0.43 |
| Male sex - no. (%) | 22,182 (48.49) | 10,176 (49.10) | 0.11 |
| Living in own house - no. (%) | 9,987 (21.83) | 4,962 (23.94) | 0.74 |
| Living in the study area less than one year - no.(%) | 20,213 (44.18) | 8,843 (42.67) | 0.56 |
| Living in household** sharing kitchen with others - no. (%) | 40,437 (88.39) | 17,588 (84.86) | 0.45 |
| Living in household with improved water source - no. (%)‡ | 2,133 (4.66) | 960 (4.63) | 0.93 |
| Living in household using treated water for drinking - no. (%)§ | 25,387 (55.49) | 10,537 (50.84) | 0.48 |
| Living in household using fixed place for waste disposal- no. (%) | 38,236 (83.58) | 15,852 (76.49) | 0.31 |
| Living in household having concrete roof - no. (%) | 6,783 (14.83) | 2,994 (14.45) | 0.88 |
| Living in house with sanitary toilet - no. (%) | 298,(0.65) | 70 (0.34) | 0.23 |
| Monthly expenditure of household – Bangladesh taka¶ | 10,030±4,758 | 9,660±4,034 | 0.18 |

* The p-values were derived by comparing the differences between the two groups adjusted for cluster effects using generalized estimating equations with the logit link function for dichotomous variables and the identity link function for dimensional variables

†Zero time was defined as the date of dose 1 for vaccinees, and at the median date of dose 1 of the cycle of vaccination in the clusters for non-vaccinees

‡ An improved water source was defined as own tap

§ Water that was boiled, filtered, or chlorinated was considered to have been treated

¶One U.S. dollar equals approximately 80 Bangladeshi taka

** A household was defined as residents living in a compound and sharing the same cooking pot.

Note: Plus–minus values are means±SD

Table S5. Sociodemographic characteristics of the individuals in the vaccine and non-vaccine arms of the study in analysis of the P75 clusters for total vaccine protection

| Variables | Intervention arm  (n=95,254) | Non-intervention arm  (n=60,196) | P value* |
| --- | --- | --- | --- |
| Age at zero time – years† | 23±15.73 | 25±15.77 | <.0001 |
| Male sex - no. (%) | 43,314 (45.47) | 29,493 (48.99) | <.0001 |
| Living in own house - no. (%) | 21,812 (22.90) | 15,523 (25.79) | 0.67 |
| Living in the study area less than one year - no.(%) | 39,481 (41.45) | 24,320 (40.40) | 0.81 |
| Living in household** sharing kitchen with others - no. (%) | 83,829 (88.01) | 49,901 (82.90) | 0.31 |
| Living in household with improved water source - no. (%)‡ | 5,019 (5.27) | 3,131 (5.20) | 0.87 |
| Living in household using treated water for drinking - no. (%)§ | 52,159 (54.76) | 31,028 (51.54) | 0.60 |
| Living in household using fixed place for waste disposal- no. (%) | 78,061 (81.95) | 46,066 (76.53) | 0.33 |
| Living in household having concrete roof - no. (%) | 12,839 (13.48) | 9,018 (14.98) | 0.56 |
| Living in house with sanitary toilet - no. (%) | 543 (0.57) | 247 (0.41) | 0.46 |
| Monthly expenditure of household – Bangladesh taka¶ | 10,017±4691 | 9,673±4003 | 0.16 |

* The P values were derived by comparing the differences between the two groups adjusted for cluster effects using generalized estimating equations with the logit link function for dichotomous variables and the identity link function for dimensional variables

†Zero time was defined as the date of dose 1 for vaccinees, and at the median date of dose 1 of the cycle of vaccination in the clusters for non-vaccinees

‡ An improved water source was defined as own tap

§ Water that was boiled, filtered, or chlorinated was considered to have been treated

¶One U.S. dollar equals approximately 80 Bangladeshi taka.

** A household was defined as residents living in a compound and sharing the same cooking pot.

Note: Plus–minus values are means±SD

Table S6. Sociodemographic characteristics of the individuals in the vaccine and non-vaccine arms of the study in analysis of the P50 clusters for total vaccine protection

| Variables | Intervention arm  (n=63,185) | Non-intervention arm  (n=39,960) | P value* |
| --- | --- | --- | --- |
| Age at zero time – years† | 23±15.76 | 25±15.81 | <.0001 |
| Male sex - no. (%) | 28,778 (45.55) | 19,594 (49.03) | <.0001 |
| Living in own house - no. (%) | 14,969 (23.69) | 10,312 (25.81) | 0.80 |
| Living in the study area less than one year - no.(%) | 25,783 (40.81) | 16,250 (40.67) | 0.97 |
| Living in household** sharing kitchen with others - no. (%) | 55,429 (87.72) | 33,357 (83.48) | 0.39 |
| Living in household with improved water source - no. (%)‡ | 3,291 (5.21) | 2,013 (5.04) | 0.80 |
| Living in household using treated water for drinking - no. (%)§ | 34,665 (54.86) | 20,485 (51.26) | 0.57 |
| Living in household using fixed place for waste disposal- no. (%) | 52,210 (82.63) | 30,795 (77.06) | 0.33 |
| Living in household having concrete roof - no. (%) | 8,752 (13.85) | 5,825 (14.58) | 0.75 |
| Living in house with sanitary toilet - no. (%) | 401 (0.63) | 162 (0.41) | 0.44 |
| Monthly expenditure of household – Bangladesh taka¶ | 10,028±4812 | 9,647±4074 | 0.14 |

* The P values were derived by comparing the differences between the two groups adjusted for cluster effects using generalized estimating equation with the logit link function for dichotomous variables and the identity link function for dimensional variables

†Zero time was defined as the date of dose 1 for vaccinees, and at the median date of dose 1 of the cycle of vaccination in the clusters for non-vaccinees

‡ An improved water source was defined as own tap

§ Water that was boiled, filtered, or chlorinated was considered to have been treated

¶One U.S. dollar equals approximately 80 Bangladeshi taka.

** A household was defined as residents living in a compound and sharing the same cooking pot.

Note: Plus–minus values are means±SD

Table S7. Sociodemographic characteristics of the individuals in the vaccine and non-vaccine arms of the study in analysis of the P75 clusters for indirect vaccine protection

| Variables | Intervention arm  (n=35,039) | Non-intervention arm  (n=61,398) | P value* |
| --- | --- | --- | --- |
| Age at zero time – years† | 25±15.86 | 24±15.98 | <.0001 |
| Male sex - no. (%) | 19,248 (54.93) | 30,103 (49.03) | <.0001 |
| Living in own house - no. (%) | 6,196 (17.68) | 15,813 (25.75) | 0.21 |
| Living in the study area less than one year - no.(%) | 18,130 (51.74) | 24,866 (40.50) | 0.0007 |
| Living in household** sharing kitchen with others - no. (%) | 31,288 (89.29) | 50,900 (82.90) | 0.25 |
| Living in household with improved water source - no. (%)‡ | 1,548 (4.42) | 3,186 (5.19) | 0.66 |
| Living in household using treated water for drinking - no. (%)§ | 19,183 (54.75) | 31,657 (51.56) | 0.75 |
| Living in household using fixed place for waste disposal- no. (%) | 29,239 (83.45) | 46,985 (76.53) | 0.24 |
| Living in household having concrete roof - no. (%) | 5,739 (16.38) | 9213 (15.01) | 0.82 |
| Living in house with sanitary toilet - no. (%) | 209 (0.60) | 255 (0.42) | 0.43 |
| Monthly expenditure of household – Bangladesh taka¶ | 9,908±5,106 | 9,657±3,998 | 0.53 |

* The P values were derived by comparing the differences between the two groups adjusted for cluster effects using generalized estimating equation with the logit link function for dichotomous variables and the identity link function for dimensional variables

†Zero time was defined as the date of dose 1 for vaccinees, and at the median date of dose 1 of the cycle of vaccination in the clusters for non-vaccinees

‡ An improved water source was defined as own tap

§ Water that was boiled, filtered, or chlorinated was considered to have been treated

¶One U.S. dollar equals approximately 80 Bangladeshi taka.

** A household was defined as residents living in a compound and sharing the same cooking pot.

Note: Plus–minus values are means±SD

Table S8. Sociodemographic characteristics of the individuals in the vaccine and non-vaccine arms of the study in analysis of the P50 clusters for indirect vaccine protection

| Variables | Intervention arm  (n=23,156) | Non-intervention arm  (n=40,758) | P value* |
| --- | --- | --- | --- |
| Age at zero time – years† | 25±15.83 | 24±16.03 | <.0001 |
| Male sex - no. (%) | 12,682 (54.77) | 20,000 (49.07) | <.0001 |
| Living in own house - no. (%) | 4,244 (18.33) | 10,497 (25.75) | 0.27 |
| Living in the study area less than one year - no.(%) | 11,891 (51.35) | 16,632 (40.81) | 0.0024 |
| Living in household** sharing kitchen with others - no. (%) | 20,662 (89.23) | 34,031 (83.50) | 0.30 |
| Living in household with improved water source - no. (%)‡ | 967 (4.18) | 2,051 (5.03) | 0.55 |
| Living in household using treated water for drinking - no. (%)§ | 12,779 (55.19) | 20,904 (51.29) | 0.69 |
| Living in household using fixed place for waste disposal- no. (%) | 19,482 (84.13) | 31,410 (77.06) | 0.26 |
| Living in household having concrete roof - no. (%) | 3,924 (16.95) | 5,946 (14.59) | 0.61 |
| Living in house with sanitary toilet - no. (%) | 157 (0.68) | 166 (0.41) | 0.37 |
| Monthly expenditure of household – Bangladesh taka¶ | 9,871±5,047 | 9,630±4,064 | 0.60 |

* The P values were derived by comparing the differences between the two groups adjusted for cluster effects using generalized estimating equation with the logit link function for dichotomous variables and the identity link function for dimensional variables

†Zero time was defined as the date of dose 1 for vaccinees, and at the median date of dose 1 of the cycle of vaccination in the clusters for non-vaccinees

‡ An improved water source was defined as own tap

§ Water that was boiled, filtered, or chlorinated was considered to have been treated

¶One U.S. dollar equals approximately 80 Bangladeshi taka.

** A household was defined as residents living in a compound and sharing the same cooking pot.

Note: Plus–minus values are means±SD
